# Thoughts on Suits for Malpractice, Suggested by Certain Judicial Proceedings in Erie County, Pennsylvania

**Published:** 1849-12

**Authors:** 


### ECLECTIC DEPARTMENT,

### AND SPIRIT OF THE MEDICAL PERIODICAL PRESS.

---

*Thoughts on Suits for Malpractice, suggested by certain Judicial Proceedings in Erie County, Pennsylvania.* By WILLIAM M. WOOD, M. D., U. S. Navy.

Since my duty has required my presence in this section of country, I have several times been summoned as a witness in trials for malpractice. The character, conduct and results of those cases, go to show that in them, as too often in others, the principles of law, intended for the protection of the community, are perverted into powerful instruments of wrong and injustice.

The general influences, leading to these perversions of justice, can be readily understood and appreciated by all, and it may not be out of place to notice them, before detailing the special cases in which these influences have been manifested. It is well known, that all efforts to limit the exercise of the profession of medicine to those who have the abilities and acquirements essential to its proper understanding, have utterly failed; and ignorant and impudent pretenders, under a great variety of humbugging titles, come before the public with equal rights, and a better chance for popular favor, than the regular practitioner. The public, unfortunately, seems to consider all efforts to limit the practice of medicine to those of scientific attainments, as the attempt of a sect to monopolize rights, and to infringe upon the largest liberty. Under this latitudinarian license, we have Indian doctors, urine doctors, root doctors, water doctors, steam doctors, and homœopaths, preying upon the community; and each has his representative here. Some among them are too ignorant to know their own ignorance; and others, cunningly calculating how much the mystic and marvellous—the disposition to be humbugged—preponderate in human nature, over plain reason and common sense, appeal for a living to the greater ingredient, and shrewdly, if not honestly, make their bread out of the folly of their neighbors; only adapting their machinery to the refinement of the classes with which they deal. Charms and amulets do for some. The beautiful, refined, and fascinating vision of mystical phials and infinitessimal doses, touch the same chord in others. But how stands the regular practitioner affected by such circumstances? Why, it is this assemblage of varied dye, which represents the profession to the community; they are all of the healing art—doctors—as well as the M. D., and the interests and prejudices of the whole class are against the acts and doings of the regular practitioner. Hence, ignorance and charlatanism become the rule, and intelligence the error. As will be seen, it may be very unsafe to cure a man on scientific principles. The anecdote of the old lady, who gave implicit belief to her sailor son's story of drawing up Pharaoh's chariot wheel on the fluke of their anchor,—but when he told of flying fish, charged him with lying, is an apt illustration of the faith and credence reposed in those who, studying nature's laws and truths, attempt to act by their direction. The course of the regular practitioner is all wrong; and, for unfortunate results, whether from the nature of the case, the neglect of his patients, or contingencies beyond his control, the regular practitioner is held responsible to a judicial tribunal, and he alone is held responsible. People have a right to die contrary to art, but, *secundem artem*—never.

For some such unfortunate case, the regular practitioner is arraigned before a jury, the whole horde of 'doctors' interested in driving him out, are summoned to give testimony. The jury is, of course, not accustomed to the mental discipline of investigating medical facts, and it may be, have the common prejudice against the regular practitioner. The most preposterous and absurd opinions are given to the jury in incomprehensible language, and with a pompous and dictatorial manner, but which makes an impression where the simplicity of truth would be unheeded. The very men who give this testimony are, perhaps, those who have instigated the suit. Add to the foregoing circumstances, the avarice of those disposed to escape their doctor's bill, and willing to take the chance of making money out of their injuries; the ingenuity of lawyers watching for cases, and determined to make fees; and it will be seen the regular practitioner pursues his profession under risk and hazards which no prudent man would encounter. The consequence is, a fearful retribution is being visited upon the community in which such persecutions are sustained. Some of the most competent young men are driven off, and such as remain refuse to take the responsibility of surgical cases. Humanity is paralyzed in its efforts to relieve suffering, for I am told that services rendered in charity have terminated in a malpractice suit.

I was informed by an intelligent lawyer that, before a jury he would feel no confidence in the correctness of the practitioners course saving him from damages. Another informed me that, when upon the bench, a suit was tried before him for malpractice, in the reduction of a dislocated shoulder. The ground was, that, at the moment of reduction, a 'click' was heard, and, since then, the shoulder had been easily dislocated. It was proved against the doctor, by medical testimony that, at the time of reduction, he had ruptured the *round ligament of the shoulder-joint*, and the jury rendered a verdict against him of five hundred dollars. The judge accidentally learned that there was no such ligament, and set aside the verdict. For such a deplorable state of affairs, deplorable for the profession, and more so for the community, the illiterate alone are not responsible. The singular support which the influence of the pulpit too often gives to visionary and quacking schemes, has ever been a subject of wonder and regret. It is this influence which enables quackery to appeal to the public in the garb of respectability, and to appear to the mass as having equal claims with science. We should suppose that the laborious students of divinity, aware of the assiduous discipline and mental training, necessary to give the power to study—the power to acquire and arrange facts, to trace their connections, to investigate speculations, and to comprehend principles—would be the last to suppose that the comprehensive and intricate principles of medical science could be intuitively acquired by ignorant men. I cannot forbear quoting the remarks, or part of them, upon this subject, from the 'Discourse of Dr. Francis, before the New York Academy of Medicine.' In allusion to the clergy, he says:—'They, with all their benevolence, superadded to the weight of their sacerdotal office, sustain theories, and give credence to alleged facts, which are often at war with the best established principles of medical science, and indirectly do harm to that calling which is like unto their own corporeal nature, demanding physical relief, as the immortal soul, the support of divine counsel.'

'The Right Rev. Bishops of the Church of Great Britain, with their subordinates, who set forth, in solemn testimony, their convictions of the cures derived from Perkins' metallic tractors, humiliating as was the spectacle, impugned not the orthodoxy of their religious faith; but it would be difficult to exonerate them from blindness in observation, and fallacy in judgment, in medical affairs.'

'I well remember,' says Dr. Francis, 'an interview I enjoyed with the late Rev. Dr. John M. Mason, the unrivalled preacher of this country in days past. He had repeatedly traveled abroad, and extensively visited Great Britain. He was asked why he did not give to the public the results of his observation. 'Alas!' he replied, 'what sort of travels can I write? I neither understand the nature of the air I breathe, nor the water I drink, nor the earth I tread upon. My life has been appropriated to polemical divinity.' The frankness of this answer was characteristic of this honest and great man, who long bore the name of the American Paul.'

The eyes of the clergy should be opened by one fact, if they are closed to reflection, and it is, that those of their body who study medicine scientifically, are never the advocates of quackery.

The bearing of the foregoing widely extended and powerful influences, will be illustrated by the following facts:—

Within a recent period, a young German physician and surgeon, Dr. B., located himself in this place, and he was one whose talents and acquirements should have secured him welcome and encouragement. With gentlemanly manners and deportment, he had a superior general education. As a pupil of Dieffenbach, he had enjoyed and profited by great professional advantages.

This gentleman informed me that, from an early period of his residence, he had been persecuted by suits or threatenings of suits, let the results of his surgical practice be what it might; the people being, as he alleged, instigated to these suits by the irregular practitioners, who regarded his advent with a jealous eye, and who were called to the witness-stand as professional witnesses when the case came on. Prosecution was threatened, not only in cases of recovery with real or imaginary defect, but when his dislocations were reduced, and his fractures united, he was threatened with proceedings for having treated cases in which there had been no injury.

I was first summoned, on his part, to give evidence in the following case:

During the prevalence of small-pox, two girls, who had been exposed to the contagion, applied to him to be vaccinated. Before the vaccine influence could manifest itself, one of the girls was taken with variola, and which proved to be a very bad case of confluent disease, leaving the girl dreadfully scarred. The arm of the other girl became very much inflamed. The charges against the doctor were, that he had communicated the small-pox in one case, and some unknown animal poison in the other. Medical men, as they called themselves, whoever they were, who advised this prosecution, ought to have known that extensive inflammation sometimes follows the insertion of the vaccine virus, and to have considered that, in the other case, the variolous disease had been communicated before the girl was vaccinated. The violence of the disease alone, would contradict the idea of its being inoculated. But, on account of these cases, Dr. B. underwent a great deal of guerrilla persecution, professional and lay, besides being compelled to enter into expenses, and to take measures to protect himself before the court. The case was first brought before arbitrators, but was withdrawn from them to be carried into court. Dr. B. wearied of vexation, having made arrangements for his defence, in his absence, left his suits, his property, and his family, to seek a more generous home and better rewards, in the golden valleys of California. Soon after the withdrawal of the foregoing case from the arbitration, I was summoned before a magistrate to give evidence in another suit against Dr. B., or rather payment of the doctor's bill was resisted, upon the defence of malpractice.

A farmer in good health, and in the prime of life, had his left leg fractured by the fall of a heavy piece of timber. The fracture was about the middle of the leg, and compound, both bones being driven through the soft parts, forming the calf. There was of course much extravasation. The doctor thought there might be a chance of saving the leg. He placed the bones in apposition, and retained them there by means of straw splints, made by neatly rolling a volume of straw in each end of a piece of canvass, and bedding the contused and fractured limb between them. The foot was prevented from falling over by a foot-piece made fast to the foot of the bed. Cold water dressings were at first applied, and afterwards a vinous wash. In the course of a few days, coagula clogging the orifices in the soft parts, and there being much stuffing of the limb from effused blood, Dr. B made one or two incisions, to free it. Gangrene came on, and the limb was amputated by the double flap operation, through the lower third of the thigh.

Respecting the time occupied in the operation, there was a difference of opinion, the longest asserted, being forty-three minutes up to the conclusion of the dressing, and part of this was lost by the operator, having scalded his hands. About two days after the operation, the man died.

Taking into consideration the great mortality attending thigh amputations, I was at some loss to conjecture the grounds for alleging malpractice, and presumed it would be charged from the delay of the amputation. This was made a point, but barely touched upon. Malpractice was attempted to be shown from all the following points:—

1. The operation not being primary.
2. The time occupied.
3. *Not bandaging and enclosing the limb in firm splints from the beginning.*
4. The application of cold water.
5. Fastening the foot to the foot-board.
6. Making incisions.

The third, fourth, fifth, and sixth points were those most urged, and a Thompsonian or Botanic physician, and a Homœopathic, gave evidence adverse to the doctor, on all these points. The scientific and unprejudiced character of the testimony may be judged, but the Thompsonian was complimented by one of the lawyers upon the excellency of his testimony.

The incisions, and the failure of the doctor to bandage this crushed and contused limb, were insisted upon as very great faults. The regular physicians sustained, on the witness-stand, the course pursued by Dr. B., and the justice rendered a verdict in his favor. What would have been the result before a jury, is by no means certain. But the exile of Dr. B. has, I believe, been the means of preventing this case from coming into the court.

Dr. B. having got out of the country, I began to hope that my services as a medical witness were, at least for the present, at an end; but, at the very next term, that of the present month, I was summoned to give evidence in the following case. A woman, by a fall through a bridge, received an injury of the right arm. A country practitioner was called, and pronounced the injury a fracture, and treated it with a patent splint, for fractured radius. After six days' attendance, this gentleman was told he need not come again unless sent for, and from that time the family treated the arm without any professional advice. It is now a year since the accident, and the fractured radius is well united, but there is a dislocation of the carpal extremity of the ulna. In this case, the jury found against the doctor four hundred and fifty dollars, which, added to the costs and loss of reputation, is, to a poor country practitioner, an overwhelming verdict.

It was not in proof, that the Dr. discovered the dislocation at the time he reduced the bone, neither was it in proof that he did not, further than that he made no mention of it.

There are some important questions suggested by this case, and of interest to the profession and to the community. Admitting that the dislocation was not discovered by the doctor during his attendance, ought he to be held responsible for the final result, when he was dismissed after six days' attendance? Had he been continued, it would have afforded him the opportunity of discovering the injury in time for its reduction, though it may be doubted whether, under any circumstanses, the recovery would have been better than it has been. But again, how far ought the common practitioners through the country to be held amenable to a standard of professional excellence, which neither the ability nor the inclination of the people will justify by pecuniary compensation, and which excellence is, moreover, difficult of attainment, unless by a greater amount of surgical practice than the country affords? These certainly are important considerations for the profession, and for the people.

The existing state of affairs, it appears to me, is worth the consideration of those who are interested in the question of medical education, now agitating the profession. Some few years ago, I heard a professor, in one of our principal medical institutions, defend, in his introductory lecture, the facility with which diplomas are granted by our colleges, upon the ground that it is better to provide the people with imperfectly educated physicians than with those not educated at all. But here we see the people repudiate such a doctrine; they not only claim to have, in all the highways and byways, the most skilful and accomplished men, but those who must make no mistakes and be liable to no accidents. It is better to be without a diploma; for then, besides having the sympathies of the community, the practitioner can say, 'I make no pretensions, I offer no certificate of ability, and only gave my neighbor, in his sufferings, such aid as I could.'

One of the most able and experienced practitioners here, now refuses to take the responsibility of surgical cases, and feels constrained to turn the applicants away, to find help where they can. Being urged to take charge of a dislocated elbow, which, for want of aid, went a week unreduced, he would consent to do so, only upon being released from all responsibility. During the progress of the cases narrated above, a pregnant lady, it is currently reported, expelled her fœtus, and she herself died under the action of repeated lobelia emetics, exhibited by a botanic physician; but no proceedings, I believe, were instituted in this case.

It would certainly be a source of congratulation, if those who tamper with the health and lives of the people, whether by criminal ignorance or wilful neglect of their duties, could be held to a healthful accountability; but it is too much to require the regular practitioner only to be responsible; and to be not only responsible for the unfortunate contingencies of his profession, but subject to the avarice of the community, the influence and opinions of charlatans, the acute management of the bar, and the ignorance of juries. Such is the case at present, and the only remedy that I see, lies with the bench. Let judges make themselves acquainted with what should be the qualifications required of medical men, according to the *standard justified by their location*, and charge juries definitely and clearly upon that point. There may be other modes of setting things right for the advantage of all parties, and I shall be most happy to see them suggested. —*American Journal of Medical Sciences.*

Erie, Pa., May 24th, 1849.

